# Corneal Stromal Stem Cell-Derived Extracellular Vesicles Attenuate ANGPTL7 Expression in the Human Trabecular Meshwork

**DOI:** 10.1167/tvst.14.1.21

**Published:** 2025-01-23

**Authors:** Faycal Moujane, Chi Zhang, Robert Knight, John Y. Lee, Sophie X. Deng, Jie J. Zheng

**Affiliations:** 1 Department of Ophthalmology, Stein Eye Institute, David Geffen School of Medicine, University of California, Los Angeles, CA, USA; 2 Department of Ophthalmology, Erasme Hospital, Université Libre de Bruxelles (ULB), Brussels, Belgium; 3 Molecular Biology Institute, University of California, Los Angeles, CA, USA

**Keywords:** stem cells, steroid glaucoma, gene expression

## Abstract

**Purpose:**

Regulating intraocular pressure (IOP), mainly via the trabecular meshwork (TM), is critical in developing glaucoma. Whereas current treatments aim to lower IOP, directly targeting the dysfunctional TM tissue for therapeutic intervention has proven challenging. In our study, we utilized Dexamethasone (Dex)-treated TM cells as a model to investigate how extracellular vesicles (EVs) from immortalized corneal stromal stem cells (imCSSCs) could influence ANGPTL7 and MYOC genes expression within TM cells.

**Methods:**

Human TM cell lines were isolated and cultured from donor corneoscleral rims. EVs were purified from imCSSC conditioned media (CM) using size exclusion chromatography and characterized by nanoparticle tracking analysis, transmission electron microscopy (TEM), and ExoView technology. TM cells were treated with either Dex alone or with EVs for 5 days. Quantitative polymerase chain reaction (PCR) was carried out to quantify the mRNA level of MYOC and ANGPTL7.

**Results:**

A notable increase in the expression levels of MYOC and ANGPTL7 genes was observed compared with untreated TM cells (control). Furthermore, upon comparing Dex-treated TM cells with those receiving both Dex and EV treatments, a statistically significant reduction in ANGPTL7 expression (*P* < 0.05) was detected.

**Conclusions:**

The present study demonstrates that imCSSCs-derived EVs can effectively decrease the expression of ANGPLT7, a gene associated with fibrosis and implicated in the abnormal elevation of IOP in patients with glaucoma.

**Translational Relevance:**

Our study shows that imCSSC-derived EVs can specifically target ANGPTL7 expression, making them a promising preclinical therapy for glaucoma.

## Introduction

Glaucoma is a complex disease characterized by progressive damage to the optic nerve and subsequent loss of vision. It is the leading cause of irreversible blindness worldwide, affecting more than 70 million people.^1^ Various risk factors include genetic predisposition, age, steroid use, and medical conditions such as diabetes and hypertension.^2^ However, the primary glaucoma risk factor is intraocular pressure (IOP), modulated by the trabecular meshwork (TM).^1^^,^^3^^,^^4^ In the angle between the iris and cornea, the TM cells play a critical role in controlling IOP by regulating the drainage of aqueous humor and filtering debris in the anterior chamber. In some instances of open-angle glaucoma, TM cells undergo phenotypic changes leading to disruption of their native function, characterized by an alteration in cell size, a reduction in the cell number, and an increase in extracellular matrix deposition (ECM).^5^^–^^7^

Current therapies for glaucoma focus on lowering IOP, but reducing IOP alone does not specifically target the underlying pathophysiologic mechanisms involved in TM. The TM cells are renewed by a population of mesenchymal stem cells (MSCs) known as TM stem cells located at the insert zone at Schwalbe’s line.^8^ Recent studies have demonstrated that when injected into the anterior chamber of mice, human TM stem cells can home to the TM and differentiate into functional TM cells.^9^ Alternatively, MSCs from various sources provide a promising approach that could directly target and modify defective TM cells, and several studies have demonstrated that these MSCs can help preserve trabecular meshwork integrity.^10^^–^^12^

Commonly used as a therapeutic agent in many diseases, MSCs have been shown to exert potent regenerative and immunomodulatory function through their paracrine activity, secreting various bioactive factors such as growth factors, cytokines, and extracellular vesicles (EVs)^13^; recent evidence suggests that EVs are primarily responsible for the therapeutic effect of MSCs and has demonstrated promising results in several other disease models.^14^ Indeed, using EVs isolated from MSC has been a rapidly emerging field with exciting potential. EVs are lipid bilayer membrane-bound vesicles secreted by all cell types and can be classified into different subtypes, including exosomes, microvesicles, and apoptotic bodies.^15^ EVs contain a mixture of soluble proteins, growth factors, cytokines, chemokines, non-coding RNAs, and lipids, acting as a vehicle for these biomolecular components that target cells and their respective microenvironment.

MSCs can be isolated and expanded from various anatomic sources, such as bone marrow, adipose tissue, and umbilical cord cells. Corneal stromal stem cells (CSSCs) are MSCs localized in the corneal limbus, and CSSC-derived EVs have demonstrated excellent regenerative and immunomodulatory properties.^16^ In mechanical corneal injury, CSSC-derived EVs can inhibit scarring and regenerate a transparent cornea in mice.^7^ Furthermore, we established and characterized an immortalized cell line from primary human CSSCs using transduction of the large T antigen of the Simian virus 40. These immortalized CSSCs (imCSSCs) provide a consistent functional source for EVs through several lineages of cell expansion.^17^ CSSCs are neural crest-derived cells located subjacent to the basement membrane in the limbus.^18^ Given their close native proximity to the TM, synonymous embryologic origin, and success in restoring native function and pathologic gene expression in other ocular disease cell types,^7^^,^^19^ we reasoned that the EVs derived from CSSCs may exert anti-glaucoma effects on the TM cells.

Several models have been established to culture and specifically stimulate TM cells, enabling the in vitro simulation of changes occurring during glaucoma.^20^ The dexamethasone (Dex) model, which induces glaucomatous change in TM cells, has been widely studied. Dex can increase IOP in vivo and has been reported to induce hallmarks of glaucoma in TM cells in vitro.^21^^,^^22^

These cells express an upregulation of various genes that modulate the intraocular pressure.^23^ The MYOC gene, which encodes for myocilin, was one of the first genes associated with glaucoma.^24^ However, the exact role of this gene remains unknown. The ANGPTL7 gene has recently been strongly related to regulating IOP homeostasis.^25^^,^^26^ This finding suggests that modulation of this gene may hold promise as a therapeutic target for glaucoma. Therefore, in this study, we aimed to analyze the effect of imCSSC-derived EVs on glaucomatous-like TM cells.

## Methods

### Primary Human TM Cell Isolation and Culture

Using the previously described method,^22^^,^^27^^,^^28^ we obtained nine independent human primary TM cell cultures isolated from six donors in compliance with the requirements of the Declaration of Helsinki. All corneoscleral rims used for this study were disease-free and considered suitable for corneal transplantation.

Corneoscleral rims were cut into quadrants using a blade. Forceps were used to remove the iris and gently expose the human TM. The human TM was carefully dissected from the sclerocorneal rim and washed thoroughly with 1X Dulbecco’s Phosphate-Buffered Saline without calcium and without magnesium (DPBS w/o Ca^++^ and Mg^++^) obtained from VWR/Avantor (Radnor, PA, USA). The human TM segments were placed under a 22 × 22 mm glass coverslip (VWR/Avantor) in a 6-well plate and incubated for approximately 30 minutes at 37°C, 5% CO_2_. Following incubation, Complete Dulbecco's Modified Eagle's Medium (DMEM-low glucose [1 g/L], with GlutaMAX (Thermo Fisher Scientific, Waltham, MA, USA), supplemented with 20% fetal bovine serum (FBS; VWR/Avantor) and 1% of antibiotic antimycotic (AA; Mediatech, Inc., Manassas, VA, USA), was added to the adhered human TM tissues in each well. This media was carefully replaced every 2 days until a monolayer of human TM cells formed and cells reached confluency.

Upon reaching confluency, typically about 5 to 7 days, the coverslip was gently lifted, and TM cells were harvested from wells and expanded in gelatin-coated flasks containing DMEM- low glucose (1 g/L), with GlutaMAX supplemented with 10% FBS and 1% AA. Cells at passage 4 were utilized for this study.

### Quantitative Polymerase Chain Reaction

TM cells were harvested after 5 days of treatment, and total mRNA was isolated from cells using the Qiagen RNeasy mini kit. The concentration of RNA was measured using the NanoDrop. Quantitative polymerase chain reaction (qPCR) was carried out using the Realplex 2 with TaqMan primers for human GAPDH, MYOC, and ANGPTL7. The qPCR data were collected in triplicate and analyzed with the delta-delta method.

### CSSC Culture

**Figure 1.**
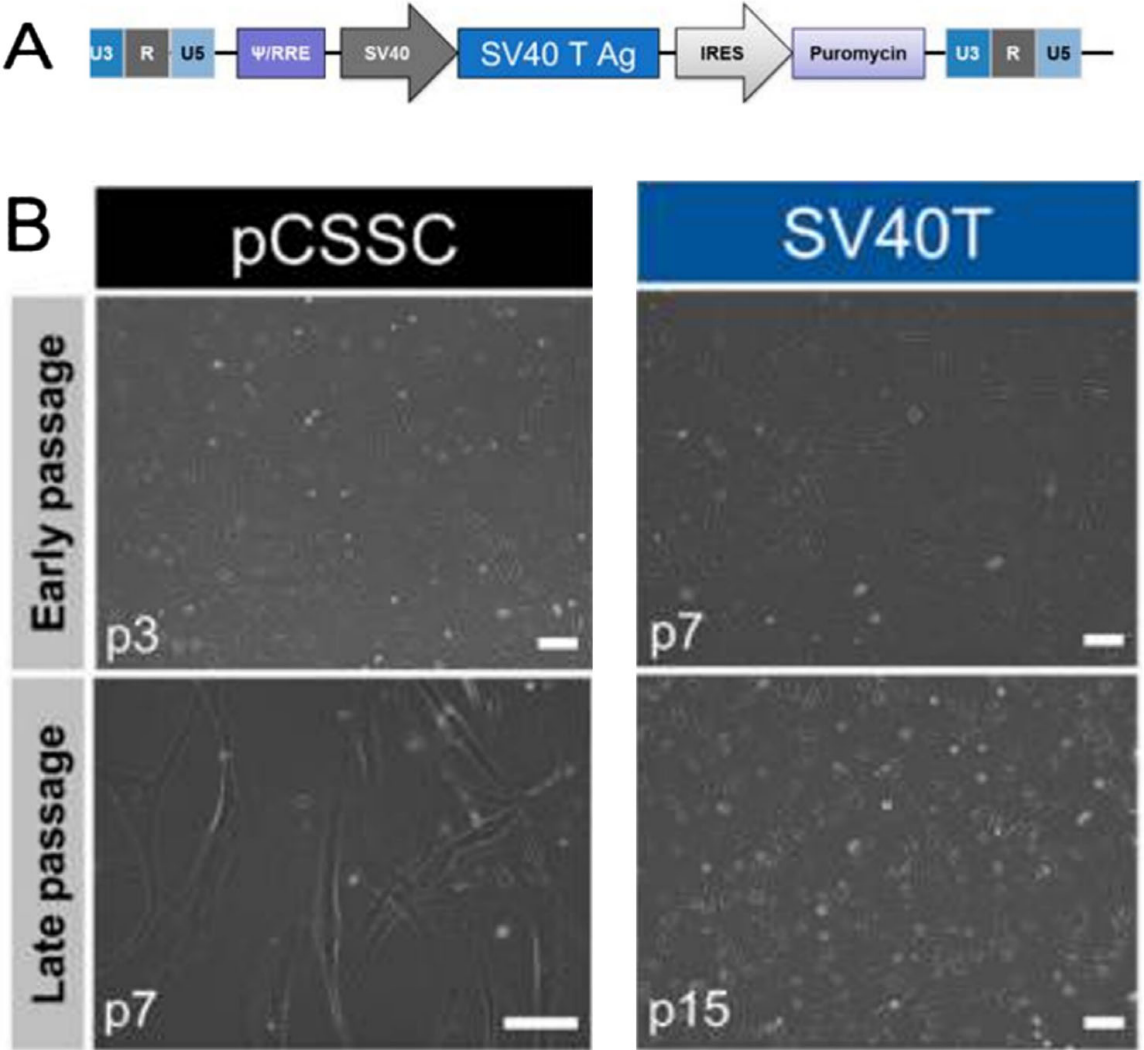
**Generation of immortalized CSSC.** (**A**) Schematic representation of lentiviral constructs for SV40T immortalizing methods. (**B**) Phase-contrast images of pCSSC and imCSSC. Scale bar = 100 µm.

The University of California, Los Angeles Institutional Review Boards evaluated and exempted the experimental protocol. The eye banks obtained consent for the tissues to be used for research. CSSCs were isolated as previously described.^29^^–^^31^ An immortalized CSSC (imCSSC) cell line was used for all experimental procedures developed and characterized previously.^17^ Briefly, primary CSSCs were transfected with Large T antigen of the Simian virus 40 (SV40T), pBSSVD2005 plasmids obtained from Addgene (Watertown, MA, USA). The SV40T gene was packaged in a lentivirus backbone with a puromycin resistance gene and transduced into primary CSSCs at passage 2. Three days post-transfection, puromycin (1 µg/mL) was added into the medium for 15 days’ selection, and the medium was renewed every 3 days (Fig. 1). The imCSSCs were cultured in CSSC medium (MCDB201/DMEM-low glucose-based medium supplemented with 2% human serum; Innovative Research, Inc., Perary Court Novi, MI), 10 ng/mL epidermal growth factor (Sigma Aldrich), 10 ng/mL platelet-derived growth factor (PDGF-BB, R&D Systems, Minneapolis, MN, USA), insulin-transferrin-selenium (ITS) solution (0.1X; Life Technologies, Carlsbad, CA, USA), 0.1 mM ascorbic acid-2-phosphate (Sigma Aldrich), 100 mM dexamethasone (Sigma Aldrich), penicillin-streptomycin solution (1X; Life Technologies), and 50 µg/mL gentamicin (Life Technologies).

### Generating CSSC-Conditioned Media

To produce conditioned media (CM) for extracellular vesicles (EVs) purification, 3 × 10^6^ imCSSCs were seeded into 5-layer T875 flasks (Corning, Corning, NY, USA) in CSSC media + 2% HS and cultured for 72 hours. After 72 hours, when cells reached 75% to 80% confluence, monolayers were washed twice with PBS, and 100 mL of serum-free CSSC media was added to cultures for 48 hours. After 48 hours, the medium was collected, centrifuged at 500 × g for 10 minutes to pellet cell debris, and filtered using 0.2 um filters. Clarified CM was stored at –80 until EV purification.

### EV Purification From Conditioned Media

Then, 100 mL frozen CM was thawed at 4°C overnight. The CM was concentrated 10 × using tangential flow filtration using a PES 100 kDa Vivaflow 50R tangential flow filtration system (Sartorius, Göttingen, Germany). The 10 × CM was concentrated a further 10 × using an Amicon Ultra-15 100 kDA filter to give 1 mL of 100 × concentrated CM. EVs were purified from 100 × CM using size exclusion chromatography. Then, 500 uL of 100 × CM was injected into a CaptoCore 700 bind elute size exclusion column, and 200 uL fractions were collected. Fraction analysis for protein concentration, particle concentration, and tetraspanin expression indicated which fractions were enriched in EVs. Fractions 4 to 8 were pooled and used as EVs in all experiments. Particle size and concentration were determined by Nanoparticle Tracking Analysis (NTA) using Nanosight NS300 (Malvern, UK).

### Transmission Electron Microscopy 

EVs were mounted on glow-discharged copper grids, 200 mesh coated with formvar carbon film (Electron Microscopy Sciences, Hatfield, PA, USA). They were fixed with 2% paraformaldehyde / 2% glutaraldehyde for 5 minutes. The grids were washed 3 times with boiled distilled water and counterstained with a 2% uranyl-oxidase solution (w/v) at pH 7 for 5 minutes. Transmission electron microscope (TEM) images were acquired using a JEOL CX100 TEM at a voltage of 60 kilovolt (kV).

### ExoView

Tetraspanin colocalization was determined using an ExoView R100 (Nanoview, Boston, MA, USA). EVs were diluted to 5 × 10^7^ particles/mL (p/mL) and incubated on EV-TETRAC chips overnight. Data are presented from four separate EV purifications.

### EVs Immunophenotyping

Then, 100 uL of a solution containing 5 × 10^9^ p/mL was added to high protein binding ELISA plates, sealed, and left overnight at room temperature (RT). The following morning, the EVs were removed, and the wells were washed using an ELISA Wash Buffer (R&D Systems, Minneapolis, MN, USA). Either PBS (intact EVs) or 0.1% Triton X-100 (permeabilized EVs) were added to the wells for 15 minutes at RT. After 15 minutes, the wells were washed with wash buffer 3 times, and unspecific antibody binding was blocked using 1% BSA for 1 hour. Then, 1 ug of primary antibody was added to each well (ABS) for 1 hour at RT. Biotin-anti-mouse antibody was diluted to 1 ug/mL and added to each well for 1 hour. The wells were washed, and 1× streptavidin-HRP was added. Substrate solutions A and B were mixed 1:1 (R&D Systems) and added to each well for 20 minutes. The reaction was stopped with the addition of 50 uL sulfuric acid. Absorbance was measured at 450 nm and 570 nm. Absorbance at 570 was subtracted from 450. Data are presented as the mean ± standard deviation from 2.

### Statistical Analysis

We first assessed the normality of the gene expression data for each condition within our samples by using the Kolmogorov-Smirnov test. Following the normality assessment, we opted to use the Wilcoxon signed-rank test for our analysis due to the nonparametric nature of our data. The Wilcoxon signed-rank test was conducted to compare the expression levels of the MYOC and ANGPTL7 genes under two different conditions: Dex and Dex combined with EVs.

## Results

### Characterization of EVs Isolated From imCSSCs

**Figure 2.**
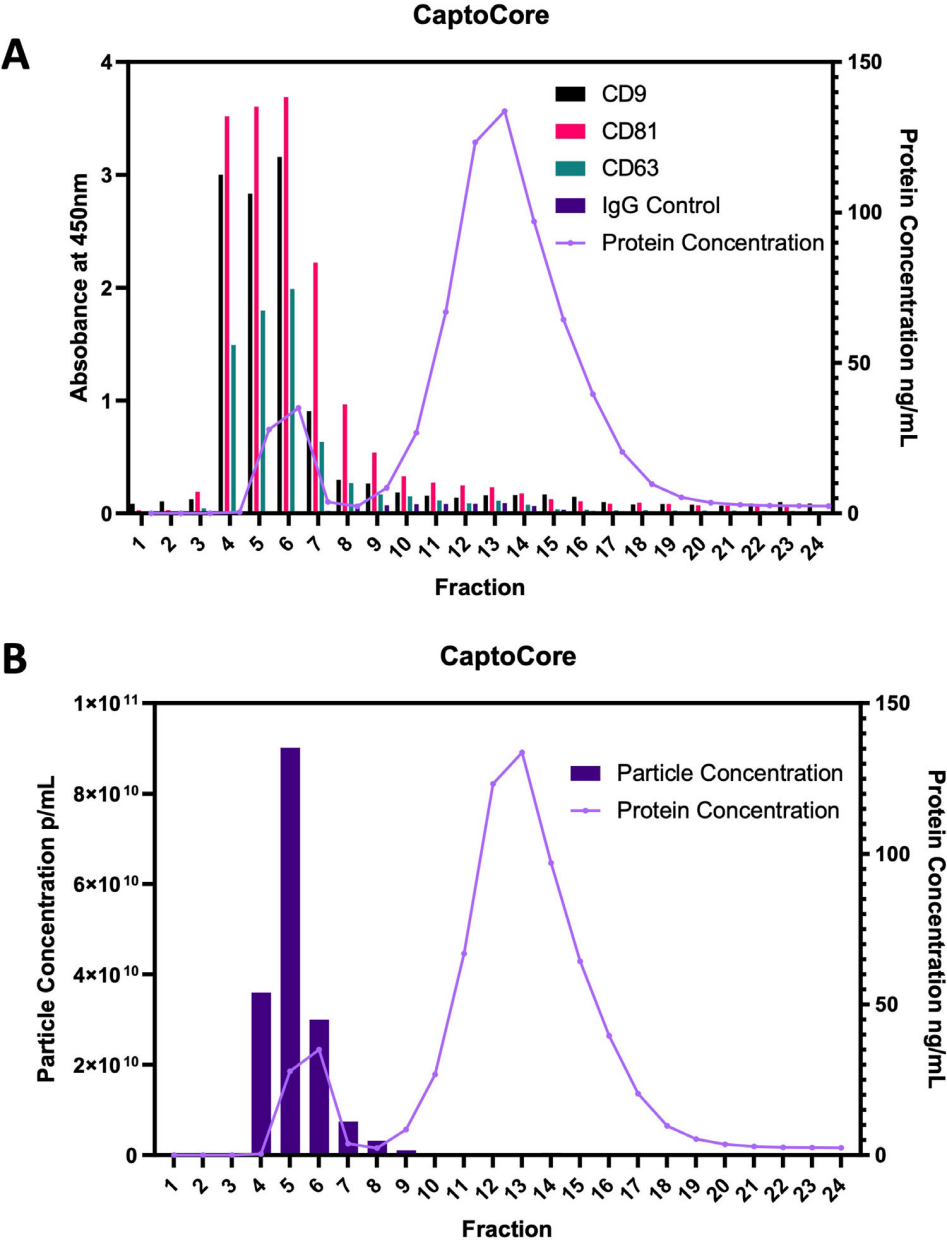
**Fraction analysis of EVs purified using CaptoCore 700 Size exclusion column*.*** (**A**) Concentrations of different biomarkers in different fractions. (**B**) EV concentrations in various fractions. Fractions 4 to 8 contain the highest particle concentration and tetraspanin abundance. These fractions were pooled together to give EVs that were used in experiments.

**Figure 3.**
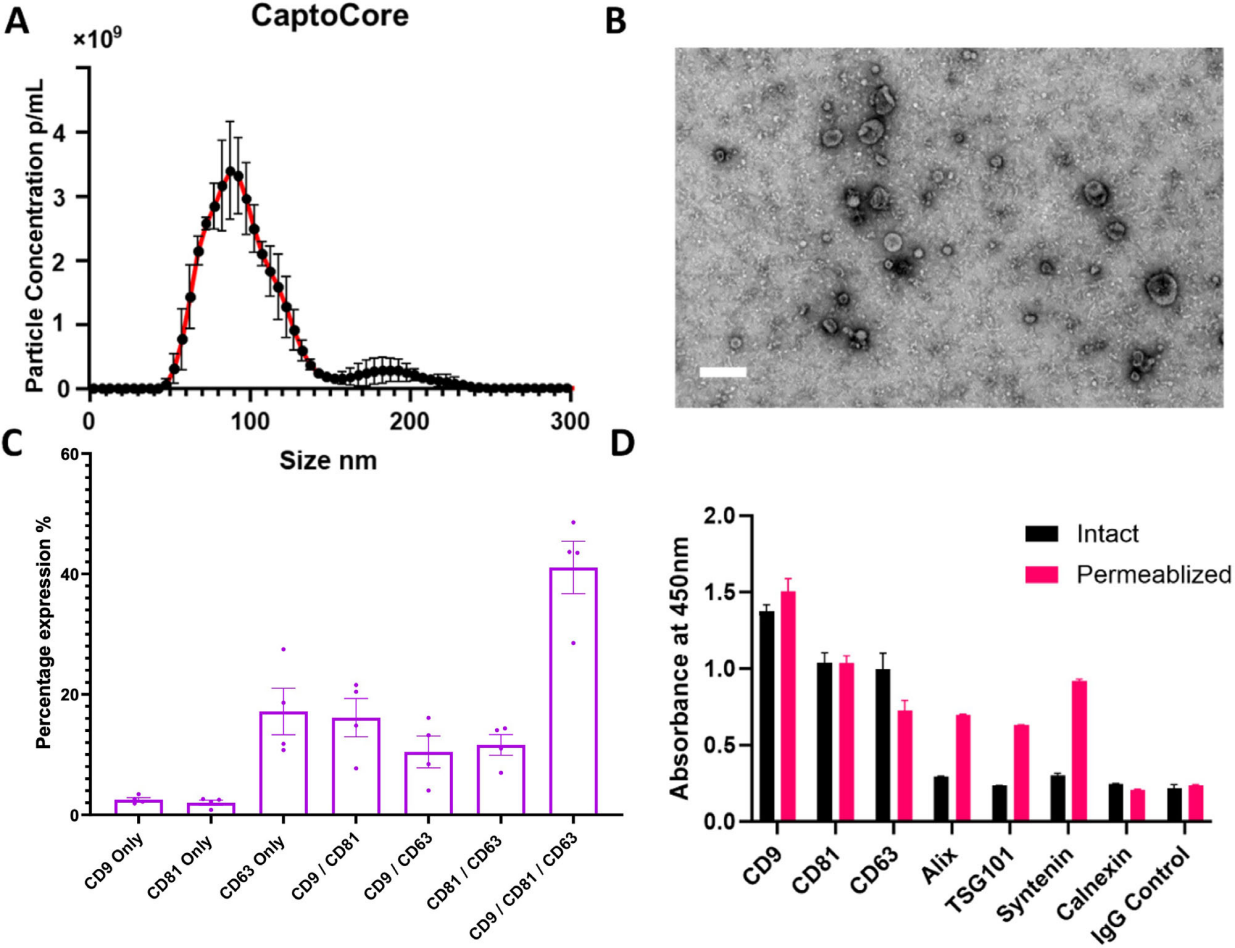
**EV characterization.** (**A**) EVs purified from imCSSC conditioned media demonstrate enrichment of particles with a model size of 88.8 nm as determined by nanoparticle tracking analysis. (**B**) TEM imaging demonstrates the presence of cup-shaped structures with a lipid bilayer, which is indicative of EVs. (**C**; n = 4) EVs contained an enrichment of tetraspanins CD9, CD81, and CD63 as determined by ExoView and (**D**) on a plate-based assay. The plate-based assay also demonstrated the intraluminal enrichment of endosomal proteins Alix, TSG101, and Syntenin-1 and the absence of cell marker Calnexin when the EVs are permeabilized (**D**). The above experiments indicate that EVs have been purified from an imCSSC-conditioned medium. Scale bar = 200 nm.

Small EVs from CM of imCSSCs were isolated by size exclusion chromatography, and the fraction analysis showed that fractions 4 to 8 contained the highest concentration of particles associated with the presence of tetraspanins CD63, CD81, and CD9 (Fig. 2). The size distribution was evaluated using NTA, indicating a mode size of 88.8 nm (see Fig. 3A). TEM revealed EVs with a cup-shaped morphology and a central depression characteristic of a lipid bilayer indicative of EVs (see Fig. 3B). The ExoView technology was used to confirm the presence of EV-specific tetraspanins CD63, CD81, and CD9 (see Fig. 3C). The immunophenotyping demonstrated the presence of specific endosomal proteins Alix, TSG101, and Syntenin-1 and the specific EVs markers mentioned before. In addition, this also demonstrated the absence of the cell marker Calnexin, which is not an EV marker (see Fig. 3D)

### Effect of Extracellular Vesicles on Dexamethasone-Induced Changes in MYOC Expression in Human Primary TM Cells

**Figure 4.**
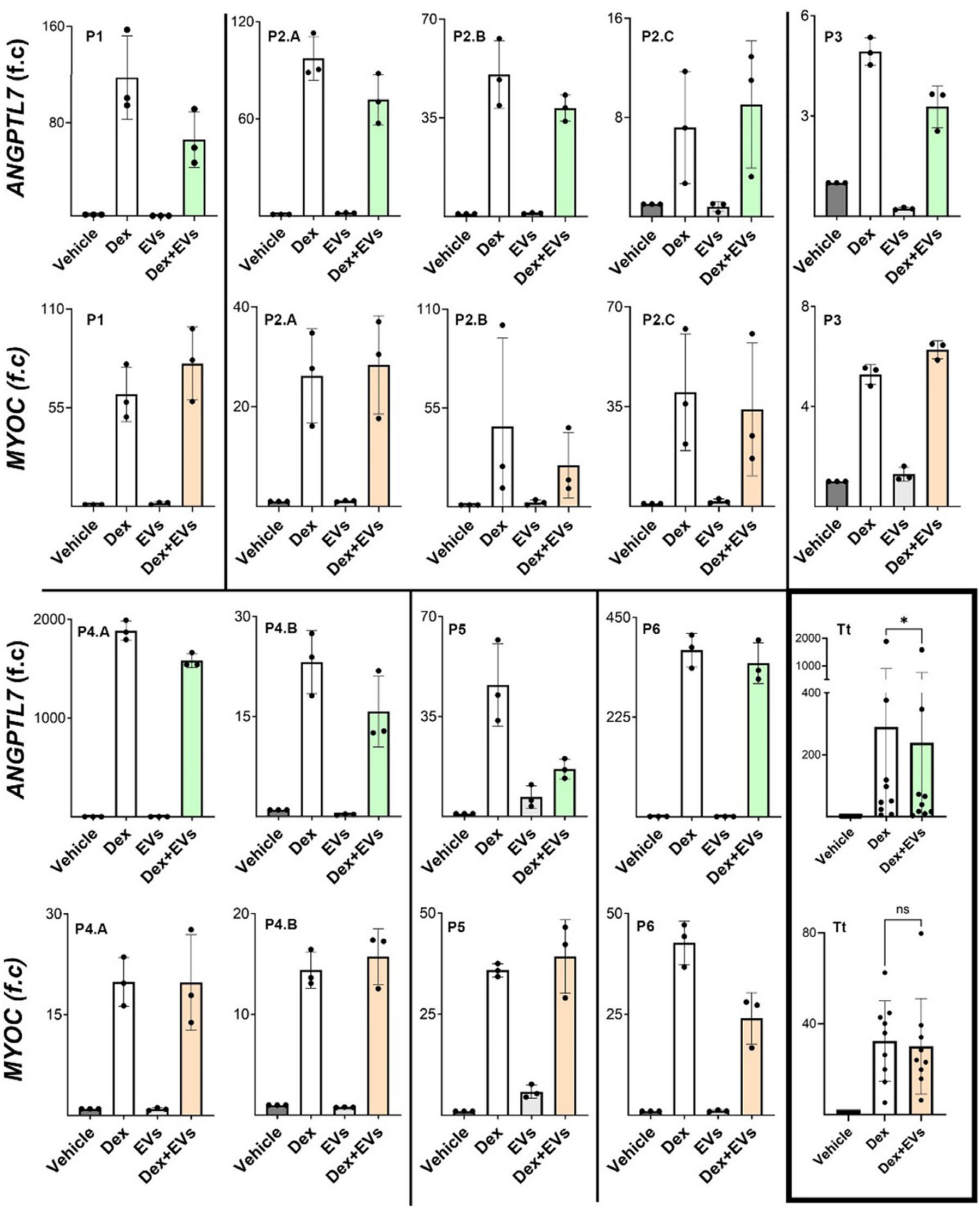
**MYOC and ANGPTL7 expression in the different groups.** Nine independent human primary TM cell cultures were established from six patients (P1–P6). The final graph includes all TM strains for each gene (Tt). TM cells were treated with the vehicle, EVs, 100 nM Dex, and Dex with EVs for 5 days. EVs only did not modify the expression of MYOC and ANGPTL7. *MYOC* and *ANGPTL7* expression was upregulated in all TM strains by Dex treatment, and EVs modulated the Dex effects. Data suggested EV has subtle/no effects on five TM strains, slightly increased *MYOC* expression on two TM strains, and significantly decreased *MYOC* expression stimulated by Dex on three TM strains. Nevertheless, no statistical significance was demonstrated. EVs suppressed *ANGPTL7* expression upregulated by Dex in 88% of TM strains and showed a significant decrease in expression levels across all samples (*P* < 0.05). We also observed that the expression levels of different genes after Dex stimulation vary widely depending on the different TM strains. This is particularly evident when analyzing the expression levels for the *ANGPTL7* gene. Indeed, 2 TM strains show a fold change greater than 300, whereas some TM strains demonstrated a fold change lower than 10.

TM cell groups were treated with vehicle, EVs alone, 100 nM Dex alone, or 100 nM Dex with EVs for 5 days, with the conditioned medium being replaced on day 3. A consistent concentration of 3 × 10^9^ p/mL was used across all the experiments. *MYOC* expression was upregulated in all TM strains after Dex treatment with an average fold of 32 (Fig. 4). Data suggested EVs have subtle/no effects on five TM strains and slightly increased *MYOC* expression in one TM strain. Nevertheless, EVs significantly decreased *MYOC* expression stimulated by Dex in three TM strains (see Fig. 4). The comparison between the Dex and Dex+EV groups across all samples does not allow for drawing statistically significant conclusions.

### ANGPTL7 Expression was Upregulated in all TM Cells After Dex Treatment and Suppressed by EVs

**Figure 5.**
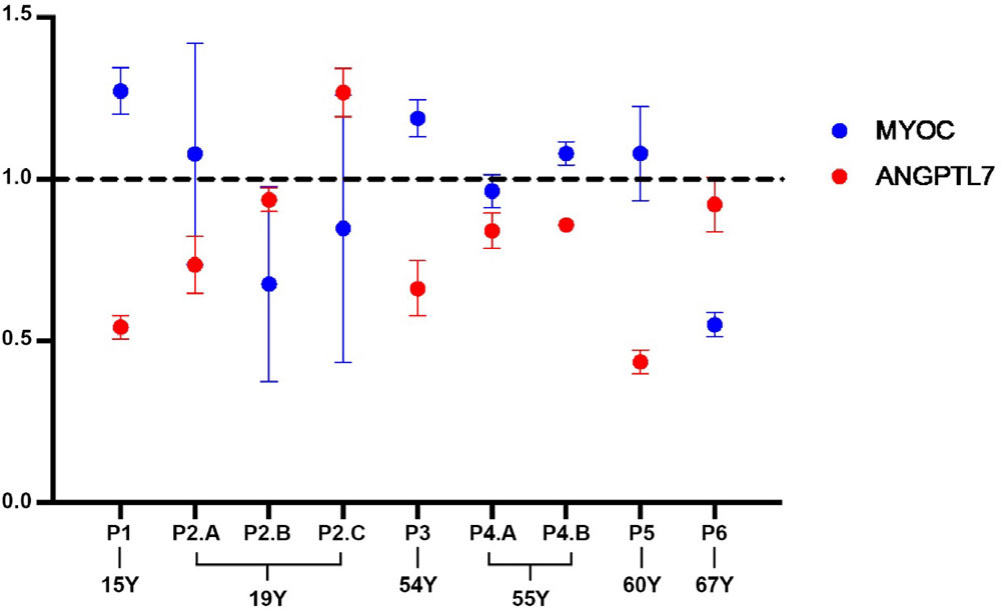
**C** **omparison between MYOC and ANGPTL7 expression in the Dex+EV group as a percentage to the Dex treatment group (% of Dex) of each TM strain.** A value above 1 indicates the EV-enhanced genes expression upregulated by Dex, whereas a value below 1 indicates the EV-suppressed genes expression upregulated by Dex. The age of the different donors was included. We do not observe a clear correlation between the expression levels of the *MYOC* and *ANGPTL7* genes for the same TM strain.

ANGPTL7 expression was upregulated in all TM strains after Dex treatment with an average fold of 290. The data suggests that EV significantly affected ANGPTL7 expression in response to Dex treatment. 88% of TM strains showed decreased ANGPTL7 expression (see Fig. 5). Furthermore, the overall *P* value was *P* < 0.05, demonstrating a significant difference in expression levels between the Dex and Dex+EV conditions across all samples. Comparison between MYOC and ANGPTL7 expression in the Dex+EV group suggests that EV had a varying effect on MYOC and ANGPTL7 expression in response to Dex treatment across different TM strains (Fig. 5). Taken together, these findings suggest that EVs can modulate the effects of Dex on gene expression in human primary TM cells, with a marked impact on the expression of the ANGPTL7 gene.

## Discussion

Although the pathogenic processes in the TM in certain types of glaucoma are not yet fully understood, we know that TM cells serve as the primary structural regulators of aqueous outflow. When the function and characteristics of these cells are altered, the aqueous humor outflow becomes impaired, increasing IOP. Moreover, we know that glucocorticoids can increase outflow resistance in the TM, leading to steroid-induced glaucoma.^32^^,^^33^ Dexamethasone can alter the expression of hundreds of genes in TM cells correlated to glaucomatous conditions.^20^^,^^23^^,^^34^ Using Dex-treated TM cells, we observed that imCSSC-derived EVs could modulate the *MYOC* gene’s expression and counteract dexamethasone’s effect on the expression of the *ANGPTL7* gene.

The *MYOC* gene was the first gene associated with glaucoma. The exact role of this gene still needs to be elucidated. MYOC expression is involved in synthesizing the protein myocilin, which can interact with other proteins in the TM, such as optineurin. It may play a role in regulating the IOP.^35^ It has also been documented that myocilin expression is more important in the TM of patients with glaucoma^36^^,^^37^ and that specific mutations in the MYOC gene can increase the risk of developing glaucoma.^35^ However, a functional study demonstrated that myocilin was not responsible for steroid-induced ocular hypertension in a mouse model.^38^ In our study, EVs derived from imCSSCs had a variable effect on the expression of the *MYOC* gene in Dex-stimulated TM cells. We observed an upregulation in one TM strain and a downregulation in three TM strains, whereas five TM strains showed no apparent variation in expression. Given the variability of our results in *MYOC* gene expression upon treatment with EVs derived from imCSSCs, combined with the current lack of knowledge regarding its involvement in glaucoma pathogenesis, it is challenging to draw any definitive conclusions.

*ANGPTL7* is one of the genes that show the highest upregulation after dexamethasone induction.^23^ The *ANGPTL7* gene was initially identified to be predominantly expressed in the eyes and more strongly in the cornea and TM.^39^ Since then, various studies have suggested that the expression of this gene is strongly associated with elevated IOP and that rare variants in the *ANGPTL7* gene could lower IOP.^40^^–^^42^ Elevated concentrations of the ANGPTL7 protein were observed in humous aqueous from patients with glaucoma.^43^ More recently, a genome-wide association analysis identified ANGPTL7 as a modulator of IOP. They also showed that increasing the level of ANGPTL7 in the mouse eye increases IOP, and decreasing the level of ANGPTL7 reduces the IOP level.^25^ Groups treated with imCSSC-derived EVs exhibited an apparent decrease in ANGPTL7 expression across TM strains in our in vitro model. Given the involvement of this gene in glaucoma pathogenesis, reducing its expression is a desirable outcome, and this result was observed when treating Dex-stimulated TM cells with imCSSC-derived EVs.

We decided to investigate a new therapeutic approach as we know that current treatments for glaucoma, primarily focusing on reducing IOP, are limited to delaying disease progression. Due to their potential to differentiate into multiple lineages and their ability to secrete paracrine factors, MSCs have already been extensively explored for their therapeutic potential in glaucoma.^44^ One study showed that human adipose stem cells could be induced in vitro to differentiate into a TM cell-like phenotype, and upon intracameral injection into mice, they were found to be home in the TM.^11^ The use of bone marrow-derived MSCs (BM-MSCs) has also been investigated. Injection of BM-MSCs into the anterior chamber of rats significantly reduced IOP in hypertensive eyes.^12^

Furthermore, Manuguerra-Gagne et al. demonstrated that injection of BM-MSCs into laser-induced hypertensive eyes of mice decreased IOP but also showed that the paracrine activity of these MSCs primarily mediated this effect.^10^ Indeed, the fact that BM-MSCs do not differentiate into TM cells in an in vitro co-culture model, their early disappearance from the TM in vivo, as well as the sustained effect on IOP reduction following secretome injection suggest that the effects are mediated through the paracrine functions of these cells. EVs have been described in recent years as an important component of MSC secretome activity. In a glaucoma disease model, MSC-derived EVs were shown to protect TM cells from oxidative stress in vitro*.*^45^

CSSC-derived EVs are now considered potential therapies for various ocular pathologies. They could accelerate corneal epithelial wound healing after mechanical injury.^19^ In another study, these EVs remarkably facilitated the restoration of corneal transparency in a mouse corneal injury model.^7^ Fibrosis is the main event leading to the development of corneal scars. Upon injury to the corneal stroma, keratocytes undergo a progressive transformation into myofibroblasts, forming scar tissue.^46^

Furthermore, this phenomenon is also observed in the pathophysiology of glaucoma. TM cells are differentiated into myofibroblasts,^47^ and it has been demonstrated that the secretome of MSCs can protect TM cells from this differentiation.^12^ Considering the anti-fibrotic efficacy of EVs derived from CSSC in the stroma associated with imCSSC, allowing for consistent production of EVs, it appears intriguing to explore the therapeutic potential of these EVs in the context of glaucoma.

In our study, imCSSC-derived EVs demonstrated a downregulation of the *ANGPTL7* gene when TM cells were stimulated with Dex. As explained above, the expression of this gene is strongly associated with glaucoma and, therefore, represents a promising potential therapeutic target. However, this study is limited as the results are based on an in vitro model. To confirm the therapeutic potential of EVs derived from CSSCs, further in vivo experiments in an animal model or in vitro experiments closer to the anatomic reality would be required. Nevertheless, this observation is promising and could pave the way for the exploration of alternative therapies for glaucomatous pathologies.
